# Effects of mouse utricle stromal tissues on hair cell induction from induced pluripotent stem cells

**DOI:** 10.1186/s12868-014-0121-7

**Published:** 2014-11-06

**Authors:** Akiko Taura, Hiroe Ohnishi, Shohei Ochi, Fumi Ebisu, Takayuki Nakagawa, Juichi Ito

**Affiliations:** Department of Otolaryngology-Head and Neck Surgery, Graduate School of Medicine, Kyoto University, Kyoto, 606-8507 Japan

**Keywords:** Hair cell-like cells, iPS cells, Mouse utricle stromal tissue

## Abstract

**Background:**

Hair cells are important for maintaining our sense of hearing and balance. However, they are difficult to regenerate in mammals once they are lost. Clarification of the molecular mechanisms underlying inner ear disorders is also impeded by the anatomical limitation of experimental access to the human inner ear. Therefore, the generation of hair cells, possibly from induced pluripotent stem (iPS) cells, is important for regenerative therapy and studies of inner ear diseases.

**Results:**

We generated hair cells from mouse iPS cells using an established stepwise induction protocol. First, iPS cells were differentiated into the ectodermal lineage by floating culture. Next, they were treated with basic fibroblast growth factor to induce otic progenitor cells. Finally, the cells were co-cultured with three kinds of mouse utricle tissues: stromal tissue, stromal tissue + sensory epithelium, and the extracellular matrix of stromal tissue. Hair cell-like cells were successfully generated from iPS cells using mouse utricle stromal tissues. However, no hair cell-like cells with hair bundle-like structures were formed using other tissues.

**Conclusions:**

Hair cell-like cells were induced from mouse iPS cells using mouse utricle stromal tissues. Certain soluble factors from mouse utricle stromal cells might be important for induction of hair cells from iPS cells.

## Background

Takahashi and Yamanaka [1] established a method for reprogramming somatic cells into induced pluripotent stem (iPS) cells. iPS cells can be easily established from individuals and are an important tool for the study of various diseases. Because of the anatomical limitations, the human inner ear is not readily accessible and there have been few pathological and molecular studies. This hindrance may impede development of treatments for inner ear diseases. By production of patient-specific inner ear cells, we can reveal disease mechanisms and develop phenotypic screenings for drug discovery. For example, we can show degenerative mechanisms in detail using iPS cells produced from patients with genetic disease. Some human disease-specific iPS cell lines have already been established and clinical research is about to begin in the areas of ophthalmology and neurology [2,3].

Inner ear disorders such as hearing loss and balance disorders are among the most common disabilities in our society and their major cause is sensory hair cell loss in the inner ear [4]. Therefore, intensive study of hair cells may lead to treatments for inner ear disorders. Consequently, proper hair cell induction from iPS cells is important for disease-specific iPS cell research. Oshima et al. [5] has previously reported the production of hair cell-like cells by stepwise induction of iPS cells using chick stromal cells. However, the induction efficiency is not very high. Therefore, a more efficient method should be developed for application to clinical research. In this study, we examined the potential of iPS cells to differentiate into hair cells for production of large numbers of these cells. First, we evaluated the efficiency of iPS cell differentiation into the otic lineage, which was developed by Oshima et al. [5]. For further differentiation into hair cells, they used chick stromal cells. Here, we used a very similar method in which three kinds of mouse utricle tissues were used instead of chick stromal cells to compare their effects on hair cell induction. Recently the majority of iPS studies have focused on human iPS cells. However, a hair cell differentiation method using human iPS cells has not been established yet and the effects of various factors on mouse iPS cells are quite different from those on human iPS cells. Therefore, in this study, we used mouse iPS cells that have established protocols for the hair cell differentiation.

## Methods

### Animals

Utricular maculae were dissected from 10 CD-1 mouse pups at postnatal day 2 (P2) (Japan SLC, Hamamatsu, Japan). The experimental protocol was approved by the Animal Research Committee of the Kyoto University Graduate School of Medicine.

### Mouse iPS cells

An iPS cell line derived from tail-tip fibroblasts (256H18) was kindly provided by Dr. Shinya Yamanaka (Kyoto University). Mouse 256H18 iPS cells were generated by retroviral transduction of transcriptional factors Kruppel-like factor 4, octamer 3/4, and sex-determining region Y-box 2 into mouse tail skin fibroblasts. These cells also carried the Discosoma red fluorescent protein (DsRed) gene driven by the cytomegalovirus early enhancer/chicken β actin promoter [6,7].

### Differentiation of iPS cells into the otic lineage

A previously reported method [5] was used for differentiation of iPS cells into the ectodermal lineage. First, iPS cells were seeded on round glass coverslips in a 4-well culture plate (Greiner Bio-One, Frickenhausen, Germany) and cultured under the D/S/I condition (Dkk-1, SIS3, and insulin-like growth factor-1) for 5 days to differentiate into the ectodermal lineage. Then, basic fibroblast growth factor (bFGF) was added as an otic inducer for the following 3 days of culture. We confirmed whether the differentiated iPS cells were induced into otic progenitor cells using an antibody against otic induction marker Pax2 (1:300; Covance, NJ, USA). The number of Pax2-positive cells was quantified in the cultures. Pax2-positive cells were counted in three randomly selected fields and averaged in each well (n = 5). Each square of the reticule was 500 μm on each side.

After differentiation into the otic progenitor cells, the cells were co-cultured with three kinds of mouse utricle tissues: stromal tissue, stromal tissue + sensory epithelium, and the extracellular matrix of stromal tissue.

### Tissue culture of vestibular maculae

**Figure 1:**
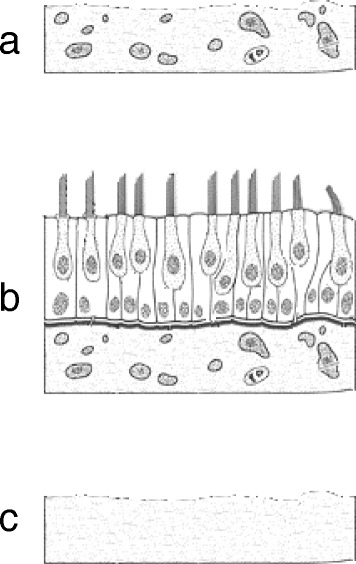
**Three kinds of utricle tissues. a**: Mouse utricle stromal tissues without sensory epithelium (MUS). **b**: Mouse utricle tissues containing sensory epithelium (MUS + SE). **c**: Extra cellular matrix after removing cellular component of stromal tissues (ECM).

CD-1 mouse pups (P2) were deeply anesthetized on ice, and then their temporal bones were removed. The organs of utricular maculae were dissected out, and immediately placed around iPS cell colonies grown in 4-well plates. The explants were maintained in Dulbecco’s modified Eagle’s medium supplemented with 10% fetal bovine serum and 30 U/ml penicillin at 37°C in a humidified atmosphere with 5% CO_2_. Each well contained about 1 × 10^4^ iPS cells in 100 μl medium and five tissue pieces of mouse vestibular maculae. The medium was exchanged every other day. The following three kinds of mouse utricle tissues were examined and each co-culture experiment was repeated at least five times (Figure 1).

#### Co-culture with mouse utricle stromal tissue (Figure 1a, MUS)

For co-cultures with mouse utricle stromal tissues, enzymatic digestion was performed with 0.5 mg/ml thermolysin (Sigma) for 5 min at 37°C. Then, utricles were divided into epithelial and stromal tissues with a 26 G needle. For hair cell induction, mouse utricle stromal tissues were the co-cultured with iPS cells.

#### Co-culture of mouse sensory epithelium and stromal tissue (Figure 1b, MUS + SE)

Co-cultures were performed for 7 days with mouse utricle tissues containing both the sensory epithelium and stromal tissue after removing the otoconia only. We placed the mouse utricle maculae in wells containing iPS cells without dividing sensory epithelium and stromal tissues.

#### Co-culture with utricle extracellular matrix (Figure 1c, ECM)

To confirm the effect of stromal cells, we removed the cellular components from the mouse utricle stromal tissues by treatment with 0.1% sodium deoxycholate (Invitrogen, CA, USA) for 1 h at 37°C. This procedure has been reported to better preserve extracellular matrix components [8]. Co-cultures were then performed with the extracellular matrix of mouse utricle stromal tissues.

The various tissues were co-cultured with the differentiated iPS cells for 7 days. Each well contained five pieces of mouse utricle tissues. The cells were subsequently cultured in normal medium that was changed every 2 days (Figure 1).

### Immunohistological analysis of hair cell induction

At the end of the culture period, explants were fixed for 15 min in 4% paraformaldehyde and permeabilized with 5% Triton X-100 in phosphate-buffered saline (PBS). After washing in PBS three times, the explants were treated with a blocking solution containing 10% bovine serum albumin. Then, the explants were incubated with a rabbit anti-myosin 7A polyclonal antibody (1:500; Proteus Bioscience, Ramona, CA, USA) followed by Alexa Fluor 488-conjugated goat anti-rabbit IgG (1:100; Invitrogen). The specimens were also incubated with Alexa Fluor 633-conjugated phalloidin (1:100; Invitrogen) for 1 h to label F-actin. The explants were then were incubated with DAPI for 15 min. Specimens were examined under a Leica TCS-SP2 laser-scanning confocal microscope (Leica Microsystems, Wetzlar, Germany).

To quantify differentiation of hair cells from iPS cells, myosin 7A and DsRed double-positive cells were counted in three randomly selected areas and averaged in each well for each condition (n = 5 − 7). Each square of the reticule was 100 μm on each side.

### Statistical analysis

Statistically significant differences were evaluated by analysis of variance and the least significant difference post-hoc test with correction for repeated measures (Stat View 5.0). P-values of less than 0.05 were considered to be statistically significant.

## Results

### Differentiation of iPS cells into the otic lineage

First, we confirmed the reproducibility of the induction method by Oshima et al. [5]. To examine differentiation of iPS cells into the otic lineage, we evaluated Pax2-positive cells. After culture under the D/S/I condition and bFGF treatment of iPS cells, there were many Pax2-positive cells. The percentage of Pax2-positive cells among the iPS cells was 11.8 ± 3.7% (SE) which is comparable to that in a previous report [5].

### Co-culture with the three kinds of mouse utricle tissues

#### Co-culture with mouse utricle stromal tissue (MUS)

**Figure 2:**
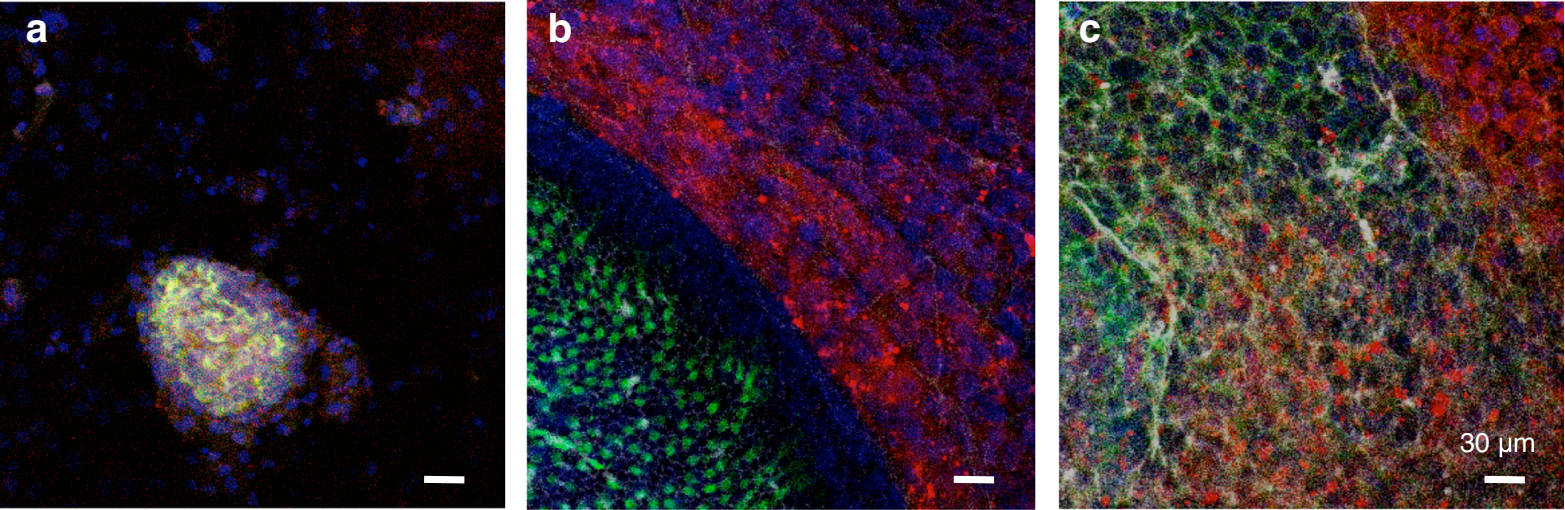
**Co-culture with the three kinds of mouse utricle tissues. a)** Co-culture with mouse utricle stromal tissues without sensory epithelium (MUS). Between DsRed-positive iPS cell colonies and stromal tissue, there were some round cell clusters containing myosin7A-positive cells and DsRed-positive iPS cells. **b)** Co-culture with mouse utricle tissues containing sensory epithelium (MUS + SE). There were many DsRed-positive iPS cells around mouse utricle tissues, but they were not adjacent to each other. **c)** Co-culture with mouse utricle extra cellular matrix (ECM). There were few myosin 7A-positive and DsRed-positive iPS cells. However, these cells were negative for phalloidin and did not form hair bundle-like structures. Green: Myosin7A; red:DsRed; white: phalloidin; blue: DAPI. Scale bars represent 30 μm.

**Figure 3:**
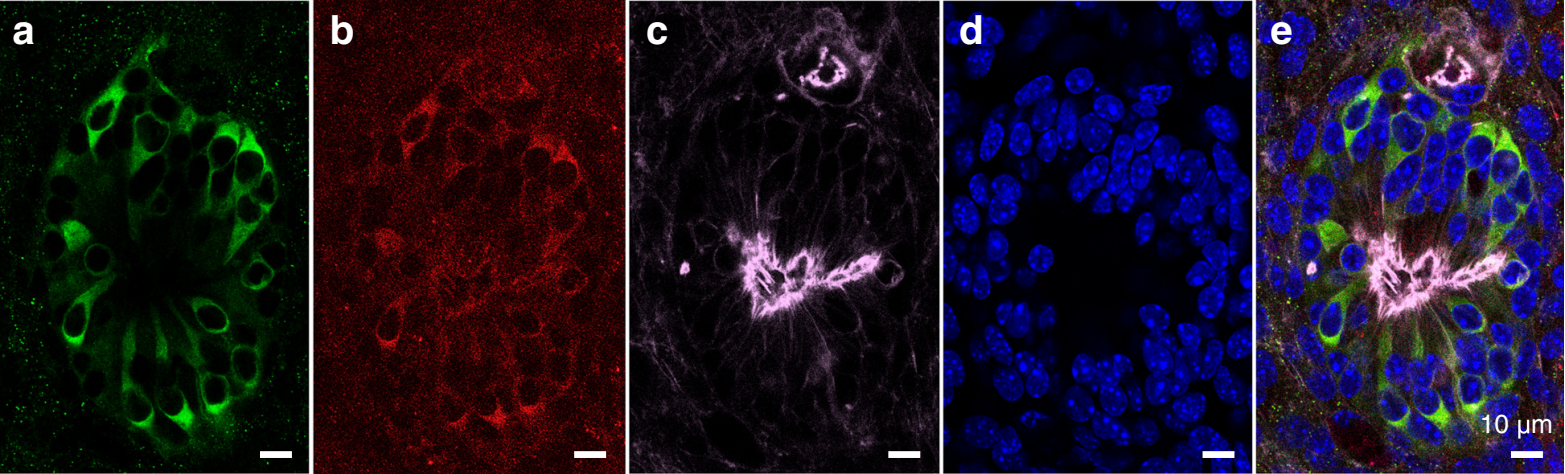
**Co-culture with mouse utricle stromal tissue (MUS).** Between iPS cells colonies and mouse utricle stromal tissue, there were some round cell clusters containing myosin7A-positive and DsRed-positive cells. Phalloidin-positive hair bundle-like structures were observed in the center of these clusters. **a)** Green, Myosin7A; **b)** red, DsRed; **c)** white, phalloidin; **d)** blue, DAPI; **e)** merged. Scale bars represent 10 μm.

Between DsRed-positive iPS cell colonies and stromal tissues, there were some round cell clusters containing myosin 7A-positive and DsRed-positive iPS cells (Figure 2a). These cells formed phalloidin-positive hair bundle-like structures in the center of the clusters (Figure 3). In these clusters, some cells were DsRed positive, but myosin 7A was negative.

#### Co-culture with mouse utricle tissue (MUS + SE)

There were many DsRed-positive iPS cells around mouse utricle tissues, but co-cultured iPS cells were distributed far from the mouse utricle tissues (Figure 2b). No myosin 7A-positive cells were observed in DsRed-positive cell colonies. Between iPS cell colonies and mouse utricle tissues, there were a few myosin 7A-positive iPS cells. However, these cells were negative for phalloidin and did not form hair bundle-like structures.

#### Co-culture with utricle extracellular matrix (ECM)

To determine which factors are important for differentiation of iPS cells into hair cells, we cultured iPS cells with the extracellular matrix of mouse utricle stromal tissues. First, we confirmed that the extracellular matrix did not contain any cells by DAPI staining. Co-cultured iPS cells with extracellular matrix only appeared to be similar to iPS cells in monoculture (Figure 2c). There were few myosin 7A-positive iPS cells. However, these cells were negative for phalloidin and did not form hair bundle-like structures.

**Figure 4:**
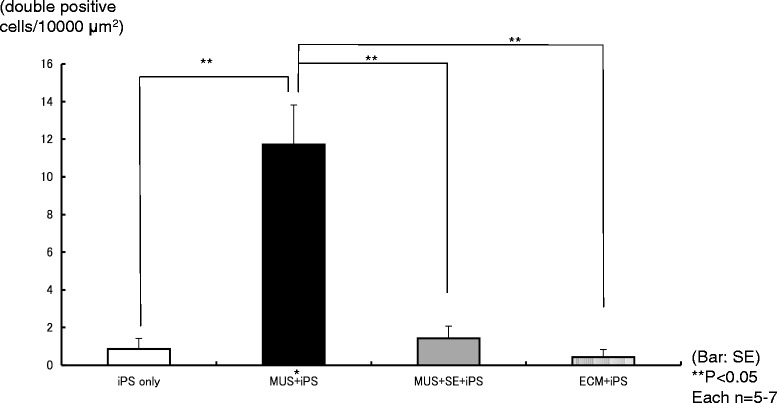
**Number of myosin 7A and DsRed double-positive cells.** Few myosin 7A and DsRed double-positive cells were observed except in MUS co-cultures that contained many double-positive cells. MUS: mouse utricle stromal tissues; SE:sensory epithelium; ECM: extra cellular matrix. Error bars represent the SE.

We compared the number of myosin 7A and DsRed double-positive cells in each condition (Figure 4). In co-cultures of iPS cells with mouse utricle tissues containing sensory epithelia, we observed few double-positive cells, but no hair bundle-like structures. Among co-cultured iPS cells with mouse utricle stromal tissues, some double-positive cells which were significantly larger than those in other conditions. The induction efficiency was 92 ± 15 (SE) cells per 1 × 10^4^ plated cells, which is comparable to a previous report [5]. In cultures of iPS cells with the extracellular matrix of stromal tissue, there were only a few double-positive cells without hair bundle-like structures.

## Discussion

First, we induced iPS cells into the ectodermal lineage by suppression of endodermal and mesodermal differentiation. Then, we differentiated the iPS cells into the otic lineage by bFGF treatment. The rate of otic progenitor cell induction was almost the same as that in a previous report [5]. After ectodermal induction, we co-cultured iPS cells with three kinds of mouse utricle tissues. Oshima et al. [5] induced hair cell-like cells from iPS cells using chicken cells. However, the induction efficiency was low and insufficient for application to clinical research. Moreover, induction of hair cells from iPS cells using mouse tissues is more appropriate because many studies of the inner ear have been performed using a mouse model. Therefore, in this study, we used mouse utricle stromal tissues instead of chicken cells. Additionally, we used mouse utricle stromal tissues without dissociating the cells to maintain the original function of the stromal cells. Because some previous reports show that the hair cells of mouse utricles can regenerate after damage, mouse utricle tissues may produce key factors that induce hair cells.

In the center of hair cell-like clusters, we observed phalloidin-positive hair bundle-like structures. Similar to a neuromast in zebrafish, all hair cell-like clusters exhibited hair bundles in their center. Therefore, our hair cells induced from iPS cells might be very primitive hair cells similar to the hair cell-like cells observed in a previous report [5].

The induction efficiency was not as high as that in a previous report, but we replaced chicken utricle stromal cells with mouse utricle stromal tissues. When we co-cultured iPS cells with mouse utricle tissues containing sensory epithelia, there were no iPS cell clusters with phalloidin-positive hair bundle like structures. This result indicates that sensory epithelia produce inhibitory factors against hair cell differentiation. According to previous reports, differentiated hair cells express Notch, and the Notch pathway inhibits differentiation of supporting cells into hair cells [9-11]. Therefore, such factors secreted from the sensory epithelium possibly act on iPS cells to inhibit differentiation into hair cells.

When we cultured iPS cells with the extracellular matrix only by removing the cellular components from mouse utricle stromal tissues, there were few myosin 7A-positive iPS cells. However, the cell clusters did not contain phalloidin-positive hair bundle-like structures. Although some extracellular matrix factors might be important but deactivated by decellularization treatments, our results indicate that stromal cells are involved in hair cell induction and some soluble factors from the cellular component of mouse utricle stromal tissues are necessary to induce hair cells from iPS cells. This finding is compatible with a previous report [5].

During cochlea development, the transcription factor Pou3f4 (Brn4) is expressed in the otic mesenchyme, which is important for coiling and lengthening of the cochlear duct and the innervation pattern of spiral ganglion neurons [12-14]. Because stromal cells exist in mesenchymal tissues, they might be related to Pou3f4, though the details have not yet been shown.

Upon detachment of the sensory epithelium, certain important factors are produced by the mouse utricle stromal cells. These factors should be identified in future studies for proper hair cell induction from iPS cells.

Our findings revealed that mouse utricle stromal tissues produce factors that induce hair cells from iPS cells, although the induction efficiency is not very high. Nevertheless, our results may help to reveal which factors induce hair cells by analyzing the differences between each condition. Comprehensive analysis of the various soluble factors secreted from mouse utricle stromal tissues may identify key factors for hair cell induction in the near future.

## Conclusions

We induced hair cell-like cells from mouse iPS cells using mouse utricle stromal tissues. Some soluble factors from mouse utricle stromal cells might be important to induce hair cells from iPS cells.
